# Brain tumor imaging of rat fresh tissue using terahertz spectroscopy

**DOI:** 10.1038/srep30124

**Published:** 2016-07-26

**Authors:** Sayuri Yamaguchi, Yasuko Fukushi, Oichi Kubota, Takeaki Itsuji, Toshihiko Ouchi, Seiji Yamamoto

**Affiliations:** 1 R&D Headquarters, Canon Inc., 3-30-2 Shimomaruko, Ohta-ku, Tokyo 146-8501, Japan; 2 Medical Photonics Research Center, Hamamatsu University School of Medicine, 1-20-1 Handayama, Higashi-ku, Hamamatsu City, Shizuoka 431-3192, Japan

## Abstract

Tumor imaging by terahertz spectroscopy of fresh tissue without dye is demonstrated using samples from a rat glioma model. The complex refractive index spectrum obtained by a reflection terahertz time-domain spectroscopy system can discriminate between normal and tumor tissues. Both the refractive index and absorption coefficient of tumor tissues are higher than those of normal tissues and can be attributed to the higher cell density and water content of the tumor region. The results of this study indicate that terahertz technology is useful for detecting brain tumor tissue.

Gliomas are the most common type of primary brain tumor and are difficult to treat. Currently, gliomas can be identified preoperatively by magnetic resonance imaging (MRI)^1^. However, the border between normal tissue and tumor tissue of gliomas is unclear, making it difficult for surgeons to remove the tumor completely. Residual tumor tissue results in recurrence of tumor growth. Additionally, opening the dura after a craniotomy changes the relative position of the brain, making it impossible to use surgical navigation because of restrictions on use of MRI during surgery. Recently, fluorescence imaging has been used to detect glioma during surgery, although it is necessary to use a fluorescent dye such as 5-aminolevulinic acid^2^. The dye must be administered preoperatively and increases the burden on the patient and sometimes stains normal tissues around the tumor. Therefore, a new method for identifying the tumor region during surgery without dye is needed.

Terahertz (THz) spectroscopy may be able to detect skin, breast, tongue, liver, and colon tumors because it can provide information on the physical properties of biological materials^3^,^4^,^5^,^6^,^7^,^8^,^9^,^10^,^11^,^12^,^13^,^14^,^15^. Main features of the THz spectroscopy are 1) high sensitivity against water content in tissues, 2) detection of variation in cells without dye, which is attributable to existence of characteristical spectrum in THz region for individual substances. The detection of gliomas using THz waves could overcome problems of above mentioned MRI and fluorescence imaging during surgery and was recently described by Son *et al.*^16^,^17^. They used the peak-to-peak reflection ratio of a THz signal to distinguish normal and tumor tissues and to generate THz images of glioma in fresh tissue from rats. Meng *et al.* demonstrated differences in the complex refractive index between normal and tumor tissues using paraffin-embedded brain glioma from mice^18^,^19^. We have reported the possibility of glioma detection from the complex refractive index obtained by THz spectroscopy of rat fresh tissue and of applying principal component analysis during statistical analysis to distinguish normal and tumor tissues^20^.

However, to our knowledge, THz imaging of gliomas in fresh tissues using complex refractive index values to distinguish normal and tumor tissues has not been demonstrated. Understanding the mechanism by which imaging can detect gliomas is important for establishing a new detection method. The mechanism can be understood quantitatively from the underlying physics by using complex refractive indices. Therefore, the purpose of this study was to investigate the possibility of using THz waves to detect brain tumors by obtaining THz images using complex refractive index values.

## Methods

### Experimental setup

A THz time-domain spectroscopy (THz-TDS) system in reflection mode was used in this study to help elucidate the mechanism responsible for differences in THz spectra between normal and tumor tissues. The details of this system have already been reported^20^,^21^. In brief, the THz wave was generated and detected using photoconductive antennas. InGaAs and GaAs grown at low temperature were used as the photoconductive layer of the generator and detector antennas, respectively. The output of a femtosecond fiber laser was separated into pump and probe beams. The pump beam was used for generation of the THz signal and the probe beam was used for detection following conversion to a second harmonic wave with a periodically poled lithium niobate crystal. A THz time-domain signal was obtained by controlling the delay between the pump and probe beams using an optical delay stage. In our setup, the peak intensity and pulse duration of the reflected THz signal from a reference sample were 45 nA and 370 fs, respectively. The signal intensity is sensitive to absorption by water vapor and was therefore improved by replacing the atmosphere in the optical path of the THz wave with dry air (almost 0% relative humidity). We can calculate the physical properties of each sample spectrum, such as the complex refractive index, from the amplitude and phase of the temporal waveform signal.

### Sample preparation and measurement protocol

We used samples from a rat glioma model to determine whether the THz method can detect glioma tissue in this preliminary study prior to clinical studies in humans. The glioma model was established by implanting C6 glioma cells into 10-week-old male Sprague-Dawley rats. All rats weighed around 300 g. Three weeks after implantation, the entire brain was removed immediately prior to THz measurements. The brain was sectioned with a slicer within 10 min of removal to avoid sample denaturation during storage, since storage of brain tissue in saline for an extended time can result in the solution penetrating into the tissue and changing the water content. Each section was carefully mounted on a flat quartz plate to maintain its shape, and a quartz cover was used to prevent drying. After the THz measurements the section was removed from the quartz plate to make a paraffin-embedded sample by fixing in a 10% formalin solution for 24 hour and embedded in paraffin. The paraffin-embedded sample was shaved by about a few hundred micrometers to obtain a flat surface and then a thin section of 3–4 μm thickness was sliced from the surface. A hematoxylin and eosin (HE)-stained image was observed using this thin section to compare to the THz image obtained prior to staining.

All experiments were performed according to the guidelines for the care and use of animals established by the Physiological Society of Japan. The experimental protocol was approved by the Ethics Review Committee for Animal Experimentation of Hamamatsu University School of Medicine.

We established a reproducible measurement protocol by standardizing all steps such as sample preparation, and measurement conditions such as the tilt and height of the sample surface because these parameters have a large effect on the THz signal in the reflection system. For example, if the sample surface is tilted, the THz wave is reflected away from the detector and the THz signal becomes weaker, and if the height of the sample surface is not precisely at the focal point, a defocused THz wave is reflected and the THz signal becomes weaker.

According to previous studies, the refractive index difference between normal and tumor tissues is at least 0.02^3^. With our protocol and system, a difference of 0.02 in the refractive index can be obtained in the frequency range 0.8 to 1.5 THz, so this frequency range was used for analysis. The frequency range was determined in accordance with the standard deviation σ_n_ of the sample measurement. We determined that a difference of 0.02 in the refractive index spectrum was observable in this frequency range when σ_n_ was less than 0.01, which is half of 0.02. It was confirmed that σ_n_ was less than 0.01 in the frequency range in the spectra of three samples of normal rat brain tissue. For the absorption coefficient, a difference of 10 cm^−1^ was observable in the same frequency range, and so the standard deviation σ_α_ was less than 5 cm^−1^.

### Data analysis

The reflected THz signal from the sample is composed of two pulses: the first pulse is from the air/quartz interface, and the second is from the quartz/section interface. We calculated the complex refractive index of the tissue section using both pulses^22^. In this calculation, the first pulse can be used as a reference, allowing the fluctuation of the THz signals to be monitored during the measurement and the pulse to be calibrated according to the fluctuations. The spectral database used in this study was constructed from the complex refractive index spectra of samples from 10 glioma model rats. Both normal and tumor regions of rat brain tissues were measured using this protocol.

We acquired the complex refractive index as a feature value from the measurements of 10 rat gliomas into a database, and a boundary was determined by linear discriminant analysis in a plot of refractive index and absorption coefficient, as described in detail below. The color of a region in the imaging data indicates the probability of the tissue being from a tumor, which is calculated from the complex refractive index of the region. The probability of a tumor is 0.5 at the boundary and becomes higher if the value of the complex refractive index of the plot is away from the boundary and toward the tumor side.

Furthermore, we attempted to generate imaging data by determining a boundary between normal and tumor regions on the score plot obtained by principal component analysis (PCA), a commonly used statistical analysis method. PCA can extract spectral features as principal components even if the peak assignment and spectral patterns are unknown and under investigation^23^. We have reported that normal and tumor regions of rat brain tissue can be discriminated with high validity by PCA^20^.

## Results

### Brain tissue spectra

**Figure 1:**
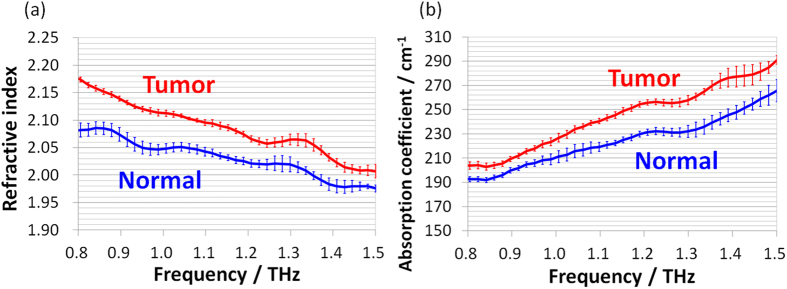
(**a**) Refractive index spectra and (**b**) absorption coefficient spectra of tumor and normal regions of a rat brain tissue sample in the frequency range from 0.8 to 1.5 THz. Red lines and blue lines correspond to the values obtained for the tumor region and the normal region, respectively.

An example of the refractive index spectra and absorption coefficient spectra of normal and tumor regions of fresh tissues from rat brain are shown in Fig. 1(a,b), respectively. In the latter sample, tumor was observed in the cortex area of the right brain and therefore we measured the same area of the left brain as normal tissue. All spectra are averaged results of five measurement points and the standard deviation is shown as error bars. One of the five measurement points was near the center of the region, and the other four measurement points were equally spaced approximately 250 μm from the central point. Peak-like features in all the spectra should be considered as artifacts generated through the calculation.

There was a spectral difference between the normal and tumor regions that was larger than the minimum observable difference in our setup, and the value of the tumor region was higher in the refractive index and absorption coefficient spectra. This tendency of the spectral difference was observed in all 10 rats.

### Terahertz imaging using complex refractive index

**Figure 2:**
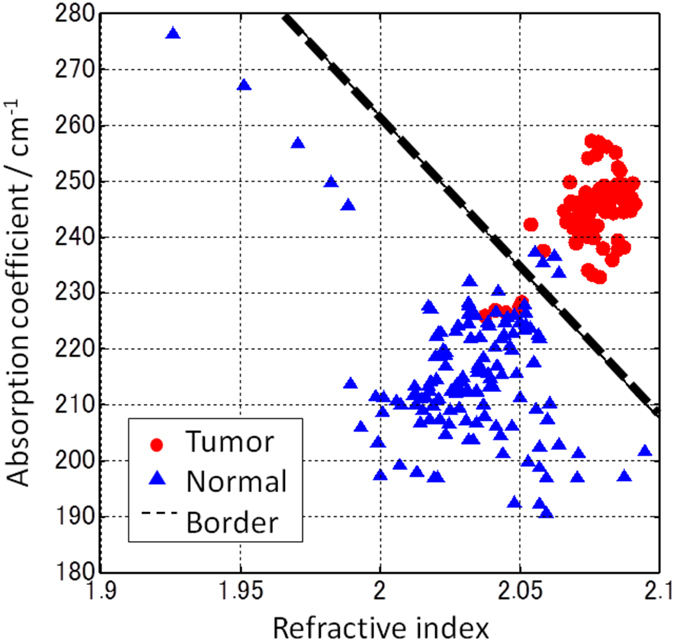
Plot of the values for refractive index and absorption coefficient obtained from 10 rats. The red circular and blue triangular points are the values obtained for the tumor and normal regions, respectively. The black dashed line shows the boundary between the normal and tumor regions.

Figure 2 is a plot of the refractive index and absorption coefficient values obtained from the spectral data from the 10 rats. The values are the average for the frequency range from 0.8 THz to 1.5 THz. A single point in the plot corresponds to the data for each measurement point of all regions of 10 rats. Five points were measured in normal and tumor regions, respectively, per rat as we mentioned already. A criterion for distinguishing normal and tumor regions was determined by linear discriminant analysis and is represented as a dashed boundary line in Fig. 2. To estimate the reliability of discrimination using this criterion, we analyzed the values of the complex refractive index at all the measurement points for the 10 rats. The total number of points correctly classified using this criterion was over 98%. This is the proportion of the calculated points classified as belonging to the same region as the measurement region after the analysis; for example, a measurement point in the tumor region was correctly classified if it was also discriminated as being in the tumor region.

**Figure 3:**
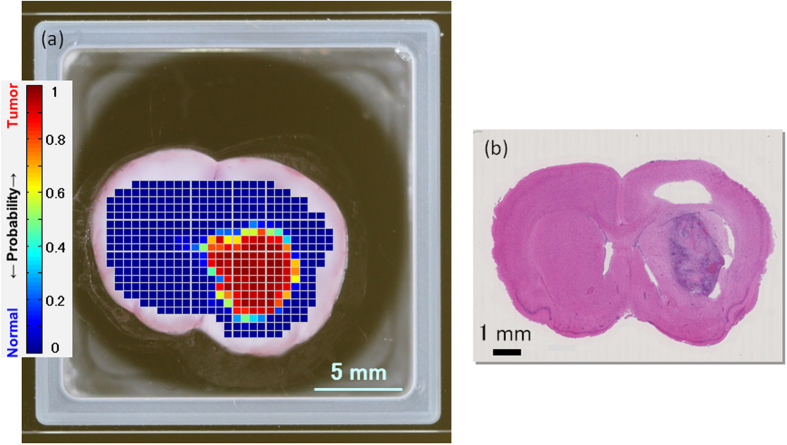
(**a**) THz image and (**b**) HE-stained image of rat brain fresh tissue.

A THz imaging result of rat fresh tissue is shown in Fig. 3(a) in which the boundary line in Fig. 2 was used as the criterion for discriminating normal from tumor tissue. The total number of measurement points in the image was 421 and the pitch was 500 μm. As mentioned above, the color of a point in the imaging data depends on the probability of tumor calculated from the complex refractive index of the region. The distribution of points in the normal and tumor regions in the plot shown in Fig. 2 could each be expressed as a normal distribution. Therefore, the probability of tumor was calculated depending on the position of the value of the complex refractive index in the plot shown in Fig. 2 and the color was determined by the probability. In the imaging data, points with high probability of being tumor are colored red and points with low probability of tumor (i.e., a high probability of being normal) are colored blue.

A HE-stained image of the same tissue section (Fig. 3(b)) shows that the region stained deep purple with hematoxylin is the tumor region. There are several white areas in the HE-stained image but these are due to the staining process and were thus not detected by the THz measurements. The THz imaging data discriminated the caudate putamen (CPu) area in the right brain as a tumor region, consistent with the tumor region in the HE-stained image.

### Terahertz imaging using principal component analysis

**Figure 4:**
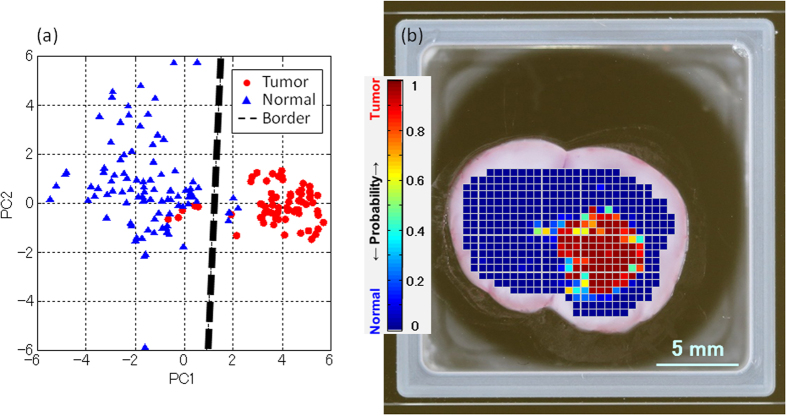
(**a**) Score plot of the PCA results expressed with PC1 and PC2. The red circular and blue triangular points are the calculation results for the tumor and normal regions, respectively. The black dashed line shows the boundary between the normal and tumor regions. (**b**) THz image colored using the results of discrimination by PCA.

We attempted to use PCA to color the THz imaging data. A score plot expressed using the extracted first principal component (PC1) and second principal component (PC2) calculated by PCA is shown in Fig. 4(a). We used six principal components for the discrimination, but a score plot of two components with especially large contributions is shown as an example. This analysis is based on spectral data similar to that shown in Fig. 1(a,b) obtained from 10 rats. Each point on the score plot reflects spectral information of both the refractive index and absorption coefficient at the same point in the tissue.

Although the points in the normal region are more scattered than the points in the tumor region, we could draw a boundary line between the normal and tumor regions by linear discriminant analysis. We could separate the normal and tumor regions according to the principal components and found that differences in the refractive index and absorption coefficient in the spectra provided the main contributions to the separation. Details of the relationship between the principal components and the physical properties of the tissue sample are currently being investigated. Consistent with the discrimination of the data shown in Fig. 2, the total number of points correctly classified was over 98%.

Figure 4(b) shows a THz imaging result in which the color is determined by the probability of tumor as calculated by PCA. The sample and measurement data are the same as that shown in Fig. 3(a). Consistent with the discrimination of the refractive index data shown in Fig. 3(a), the CPu area in the right brain was discriminated as a tumor region in the THz imaging data. Comparison of Fig. 4(b) with the THz imaging data in Fig. 3(a) suggests heterogeneity in the tumor probability in the tumor region using the THz imaging data in Fig. 4(b). The THz image shown in Fig. 3(a) is colored according to the values of the complex refractive index, whereas the THz image shown in Fig. 4(b) is colored according to the values of the score obtained by PCA and thus reflects components other than the value of the complex refractive index.

## Discussion

There are two likely reasons for spectral difference of brain tissues between the normal and tumor regions as shown in Fig. 1. First, there were differences in the cell density between the normal and tumor regions. The number of cell nuclei is greater in the tumor region than in the normal region per unit area due to the rapid proliferation of tumor cells, leading to a higher number of cells. The cell nucleus is denser than the other components because it contains many nucleic acids, which have high molecular mass and are densely packed. In general, materials with a high density have a high refractive index, and thus the higher refractive index of the tumor region is consistent with higher cell density. Secondly, the water content of the tumor region is higher because angiogenesis occurs to compensate for the lack of oxygen and nutrients. As we previously reported, water has a higher refractive index and absorption coefficient than brain tissue does, and these parameters for tumor regions are higher than those for normal regions^20^. This means that the water content is higher in the tumor region than in the normal region, and that the spectral difference between the normal and tumor regions reflects the difference in water content. A report describing the quantitative interpretation of these parameters is in progress.

THz imaging results described in Figs 3(a) and 4(b) indicated that THz imaging can detect brain tumor. Using complex refractive index resulting in Fig. 3(b), the THz imaging data could discriminate the tumor region correctly because of the higher water content and cell density in the tumor region, resulting in high refractive index and high absorption coefficient as described above. On the other side in the case of Fig. 4(b), PCA can extract spectral components as principal components and it is possible that THz imaging by PCA reflects differences between tumor regions. Although both of complex refractive index based image and PCA based image work as the tumor imaging, we should examine in detail the origin of the color difference for each analysis method in the future.

The size of the tumor region in the THz imaging data in Fig. 3(a) is larger than that in the HE-stained image in Fig. 3(b), perhaps because THz imaging reflects perifocal edema around the tumor region where there is high water content both inside and outside the cells but cell density is essentially normal. In the case of a brain tumor, pathological changes in cellular metabolism and/or the blood-brain barrier result in edema around the tumor region. In the HE-stained image, the area around the deep purple area is slightly paler in color than the same area in the left brain and this is characteristic of edema. Therefore, THz imaging may be able to detect edema around the tumor more clearly than HE staining does.

There are other possible reasons for the larger tumor region in the THz imaging data compared with the HE-stained image. One is the surface differences between the section used for the THz image and for the HE-stained image. The section is essentially the same for both images but the surface differs by about a few hundred micrometers because of the slicing process explained in the section of sample preparation and measurement protocol, which could be enough to change the position and size of the tumor. The other reason is related to the beam diameter of the THz wave, which is about 1 mm at 1 THz in our setup. At this beam diameter, both normal and tumor regions are simultaneously irradiated by the beam that means macroscopic imaging, possibly resulting in the normal region being identified as a tumor region according to the proportion of each area irradiated.

At the border between normal and tumor regions in THz imaging there is a transition band where probability of tumor changes from one to zero, which means orange, yellow and green regions in Fig. 3(a). This band might indicate areas with low burden of disease including an infiltrative pattern of dissemination around primary tumor, reflecting rate of tumor cells against normal cells due to macroscopic image of THz imaging feature. Detection of this border area is very important to avoid residual tumor tissue during the surgical restriction and after the removal of the gross tumor disease. Evaluation of a residue of the infiltrative pattern of dissemination during surgery is also important for decision of postoperative adjuvant therapy to increase healing effect. Since THz imaging has a possibility to detect this border of glioma, improved methodologies will be provided if this is verified.

Future clinical research should be required for the border detection as well as diagnosis of a grade of tumor. Since THz imaging reflects a state of substances, this modality could detect the grade of tumor by understanding the characterization of C6 glioma cells against THz wave. Furthermore if high power THz sources which match with specific vibration frequency of biological tissues are developed, THz wave will operate as a selective photodynamic or thermo- therapy. At the next step we will develop THz imaging system to be useful for above mentioned applications with additional studies and improvement of the imaging systems.

## Conclusions

We could visualize the tumor region of rat brain fresh tissue by using the complex refractive index and PCA in the THz region, thereby demonstrating that THz technology is useful for detecting brain tumor tissue. THz imaging using refractive index and absorption coefficient values may be able to visualize perifocal edema and areas with low burden of disease around the tumor region of tissues. If verified, this could be a significant advantage of THz imaging as it is difficult to discriminate a border between normal and tumor regions clearly using conventional MRI and fluorescence images during surgery. To verify this possibility, we will investigate characterization of C6 glioma cells against THz wave and compare THz imaging data with other imaging data.

## Additional Information

**How to cite this article**: Yamaguchi, S. *et al.* Brain tumor imaging of rat fresh tissue using terahertz spectroscopy. *Sci. Rep.* **6**, 30124; doi: 10.1038/srep30124 (2016).
